# Determinants of information provided by anaesthesiologists to relatives of patients during surgical procedures

**DOI:** 10.1016/j.bjao.2023.100205

**Published:** 2023-06-28

**Authors:** Audrey De Jong, Clara Penne, Natacha Kapandji, Maha Touaibia, Chahir Laatar, Michaela Penne, Julie Carr, Yvan Pouzeratte, Samir Jaber

**Affiliations:** 1 PhyMedExp, University of Montpellier, INSERM, CNRS, CHU Montpellier, France; 2 Département d’Anesthésie-Réanimation, Hôpital Saint-Eloi, Montpellier, France; 3 GRC 29, AP-HP, DMU DREAM, Department of Anesthesiology and Critical Care, Pitié-Salpêtrière Hospital, Sorbonne University, Paris, France

**Keywords:** anaesthesia, communication, family, information, intraoperative, operating room, relatives, surgery

## Abstract

**Background:**

Data and interventions are lacking for family-centred perioperative care in adults. Perioperative information given to relatives by nurses or surgeons is associated with improved satisfaction and fewer symptoms of anxiety for relatives and the patient themselves. However, the frequency of the provision of information by anaesthesiologists to patients' relatives during surgery has never been reported.

**Methods:**

A cross-sectional survey was sent to French anaesthesiologists in October 2020 to inquire how often they provided information to patients' family members during surgery and what factors led to them providing information frequently (i.e. in more than half of cases).

**Results:**

Among 607 anaesthesiologists, 53% (319/607) were male, with median age 47 (36–60) yr and nearly half (43%, 260/607) reported more than 20 years of clinical experience; most responders (96%, 580/607) mainly treated adults. Forty-nine (8%) anaesthesiologists declared that they frequently provide information to relatives during surgery. After multivariate analysis, age >50 yr, female gender, and paediatric practice were associated with providing information more frequently. Reasons for not providing information included a lack of time and dedicated space to talk to relatives. Urgent surgery or surgery lasting >2 h were identified as factors associated with provision of information to relatives.

**Conclusions:**

Giving information to relatives during surgery is not a common practice among anaesthesiologists. It depends on individual anaesthesiologists' personal characteristics and practice. Information during surgery could be provided systematically in situations identified as being the most important by anaesthesiologists in our survey. By creating new pathways of information, we could reduce stress and anxiety of patients and relatives.

Family-centred care is increasingly studied and optimised in the intensive care unit (ICU).^1^, ^2^ However, data and interventions are lacking for family-centred perioperative care in adults.^3^, ^4^ Perioperative communication and attentiveness to the patient and to relatives are two of the most important determinants of patient satisfaction.^5^ Furthermore, good perioperative communication is associated with lower anxiety and improved overall experience.^6^

However, so far, studies reporting communication with relatives during surgery have focused on information provided by nurses or surgeons.^7^^,^^8^ The anaesthesiologist's role in this context is unclear, and information by the anaesthesiologist is mostly provided before surgery, during the anaesthesia consultation.^9^ A systematic review found that communication in anaesthesia is dominated by anaesthetic planning and discussion of logistics before surgery.^7^ The two other medical specialties that routinely encounter surgical patients, namely surgery and critical care, have studied communication extensively compared with anaesthesiology.^10^, ^11^, ^12^

Patients are often supported by a family member during the course of their medical treatment. They receive the same information and may ask questions during the anaesthesia consultation. Communication with relatives is part of holistic patient care and seeks to improve understanding, satisfaction, and reduce anxiety for relatives and the patient themself.^12^^,^^13^ Moreover, relatives' satisfaction is considered a major criterion in the assessment of quality of care and of compliance with accreditation requirements.^14^

To our knowledge, there is no study reporting how frequently information and its components are given by the anaesthesiologist to relatives during surgery. The main objective of this survey was to determine how often anaesthesiologists provide information to family members, at least once, while the patient is undergoing surgery. Other important objectives were to identify the factors associated with providing information frequently (in more than half of cases), the characteristics of the information provided, and the reasons for giving information or not.

## Methods

### Study design

The I-POP (Information Péri-OPératoire) study was an institutional survey, based on best currently available evidence, guidelines, and expert opinions to identify how often anaesthesiologists provide information to patients' family members during surgery. A group of anaesthesiologists (steering committee members: CP, ADJ, YP, SJ) considered all potential items to include in the questionnaire after reviewing the literature and participating in focus-group sessions. Item reduction was performed during these sessions, resulting in a self-applied anonymised electronic questionnaire with 29 questions (Supplementary Material 1). The online survey was distributed electronically via email to French anaesthesiologist members of the French Society of Anaesthesia and Intensive Care (SFAR, Société Française d’Anesthésie Réanimation). The survey was performed in October 2020 using an electronic web-based platform (Google Forms, Google LLC, Mountain View, CA, USA). The survey was open, voluntary and anonymous and neither participants nor researchers received any compensation for taking part. There was no planned limit to the number of participants.

Pre-testing was carried out using the ‘thinking aloud’ technique (in which respondents are asked to verbalise thoughts while answering a question) to ensure adequate understanding of it.^15^ Pilot testing was performed to assure validity and, in this phase, anaesthesiologists with experience in clinical research were asked to answer all questions using an internet survey format. Questions deemed unnecessary or challenging to understand were rewritten or eliminated. Each question's response time was recorded, and questions that took more than 1 min to complete were rewritten.

This survey study did not require formal agreement because it was assumed that each participant's voluntary completion of the questionnaire constituted consent. A Checklist for Reporting Results of Internet E-Surveys (CHERRIES)^16^ was used to report the data.

### Data collected and definitions

The survey included respondent characteristics, ‘check all that apply’ or single-choice questions and open questions. They were neither randomised nor alternated. In some multiple-choice tests, more than one answer could be selected. The aim was to detail responders' own usual practice. ‘Check all that apply’ questions could be answered with one of these four choices: ‘Never (0% of cases)’; ‘Sometimes (<50% of cases)’; ‘Often (≥50% of cases)’; ‘Always (100% of cases)’.

The questionnaire is presented in Supplementary Material 1. The survey was divided in three main parts: (1) anaesthesiologist's personal characteristics and professional practice, (2) information provided by the physician to relatives before surgery, (3) information provided by the physician to relatives during surgery. The first part contained seven questions. Respondents were asked personal information (age, gender) and professional characteristics (experience, function, structure of practice, region, and main field of activity). In the second part, eight questions focused on anaesthesiologists' practices regarding information given to relatives before surgery: frequency, conditions, place, main topics, and further questions raised by relatives. In the third part, we detailed in 14 questions information given to relatives during surgery, its frequency, conditions, place, time, and main topics.

We defined the period during surgery as the period from the patient entering the operative theatre to the exit from the post-anaesthesia care unit (PACU).

The eligible respondents were defined as French anaesthesiologist members of the French Society of Anaesthesia and Intensive Care (SFAR) who work in operating theatres. The number of eligible respondents in October 2020 was estimated to be 1500.

### Statistical analysis

Responses to survey questions were analysed as one group. The data were exported and checked using Microsoft Excel (v.16.5, Redmond, WA, USA). Any errors or missing data were verified. Continuous variables were expressed as means (standard deviations) or medians (inter-quartile ranges), as appropriate. Categorical variables were summarised as number (percentages). Comparisons of proportions between groups were done using the χ^2^ test. Comparisons of continuous variables between groups were made using the Student *t*-test or Wilcoxon rank-sum test, as appropriate. Age, gender (variables judged clinically relevant), and all variables associated with providing information during surgery frequently (in more than half of cases) at a 20% threshold by logistic regression in univariate analysis were included in multivariate logistic regression, and a *P*-value-based backward selection was performed.^17^ In the final model, only significant variables were retained. Odds ratios with corresponding 95% confidence intervals were computed. Interactions between variables were tested.^17^ Missing data are described in the tables and no imputations were made.^18^ The additional free-text responses were examined using a method of thematic analysis for trends and categorised by two authors. As it was a survey, no sample size calculation was performed.

All reported *P*-values were two-sided, and statistical significance was set at *P*<0.05. All analyses were performed using statistical software (SAS Enterprise Guide, version 7.13; SAS Institute; Cary, NC, USA).

## Results

### Responders' characteristics

Survey responses were received from 607 out of an estimated 1500 eligible anaesthesiologists representing a 40% response rate. Male responders accounted for 53% of completed surveys, median age was 47 (36–60) yr, and most responders (96%) had an adult-based main field of activity. Nearly half (43%) reported >20 yr of clinical experience.

### Frequency of providing information to relatives

Forty-nine (8%) anaesthesiologists declared that they speak with relatives frequently (in more than half of cases) during the surgery: of these, 3% (21/607) declared always giving relatives information during surgery (100% of cases).

Anaesthesiologists' characteristics related to providing information in more or less than half of cases are described in Table 1. Factors associated with providing information during surgery frequently in univariate analysis were: older age (*P*<0.001), greater experience (*P*=0.027), having a paediatric practice (*P*=0.03), providing information before surgery frequently (*P*<0.001), and having a relative identified in the medical record (*P*=0.027). After multivariate analysis, three factors were associated with an increased probability of giving information: age >50 yr, female gender, and paediatric practice, as shown in Table 2.

**Table 1:** Factors associated with providing information during surgery more frequently to relatives in univariate analysis.

Characteristics	Information in >50% of cases *n*=49	Information in <50% of cases *n*=558	*P*-value
Age	55 (44–62)	46 (35–59)	<0.001
Age >50 yr, *n* (%)	31 (63)	226 (41)	0.002
Male gender, *n* (%)	22 (45)	297 (53)	0.263
Structure of exercise, *n* (%)			0.969
Teaching hospital	20 (41)	216 (39)	
General hospital	9 (18)	117 (21)	
Medical clinic	17 (35)	186 (33)	
Other	3 (6)	39 (7)	
Function, *n* (%)			0.062
Resident	0 (0)	35 (6)	
Senior doctor/assistant	0 (0)	43 (7)	
Attached practitioner	2 (4)	20 (4)	
Hospital practitioner	29 (59)	257 (46)	
Senior lecturer	0 (0)	4 (1)	
University professor	1 (2)	4 (1)	
Private practitioner	17 (35)	195 (35)	
Experience, *n* (%)			0.027
Resident	0 (0)	35 (6)	
Anaesthesiologist <5 yr	4 (8)	96 (17)	
Anaesthesiologist 5–10 yr	9 (18)	88 (16)	
Anaesthesiologist 10–20 yr	6 (12)	109 (20)	
Anaesthesiologist >20 yr	30 (61)	230 (41)	
Activity field, *n* (%)			
Adult >50%	34 (69)	477 (85)	<0.001
Paediatrics >50%	15 (31)	81 (15)	0.003
Information frequency before surgery, *n* (%)			<0.001
Never	0 (0)	14 (3)	
Sometimes	10 (20)	337 (60)	
Often	21 (43)	167 (30)	
Always	18 (37)	40 (7)	
Relative identified in the medical record, *n* (%)			0.027
Never	4 (8)	59 (11)	
Sometimes	5 (10)	153 (27)	
Often	8 (17)	93 (17)	
Always	32 (65)	253 (45)

**Table 2:** Factors associated with providing information during surgery more frequently to relatives in multivariate analysis. 95% CI, 95% confidence interval; OR, odds ratio.

Characteristics	OR	95% CI	*P*-value
Age >50 yr	2.830	1.5–5.3	<0.01
Female gender	1.880	1.0–3.5	0.047
Paediatric practice	2.487	1.3–4.8	<0.01

### Type of information provided to relatives by anaesthesiologists

Anaesthesiologists declared that providing information together with the surgical team was not common for 87% of them.

Information was most often given via a phone call (62%) or a meeting in the patient's room (53%). It could also be a meeting in a corridor (40%), in a dedicated office (28%), or in a waiting room (24%).

Moments when the physician took time to contact relatives were most commonly the occurrence of a complication (70%), and transfer from the operating theatre to PACU (55%).

The most frequently covered topics by anaesthesiologists in the absence of complication were: patient's clinical status (69%), patient's postoperative ward (50%), and information about the expected time of completion of surgery and discharge from the PACU (40%). The progress of the surgical procedure (17%) and information about a waiting room (14%) were less frequent topics.

Relatives most frequently asked about: the surgery (surgical procedure itself, 65%, and duration of surgery, 59%) and also postoperative issues (potential complications, 56%, postoperative period, 54%, and length of stay in PACU, 53%). The type of anaesthesia and potential complications were less frequent questions (29% of cases).

### Reasons for speaking to relatives

**Fig 1:**
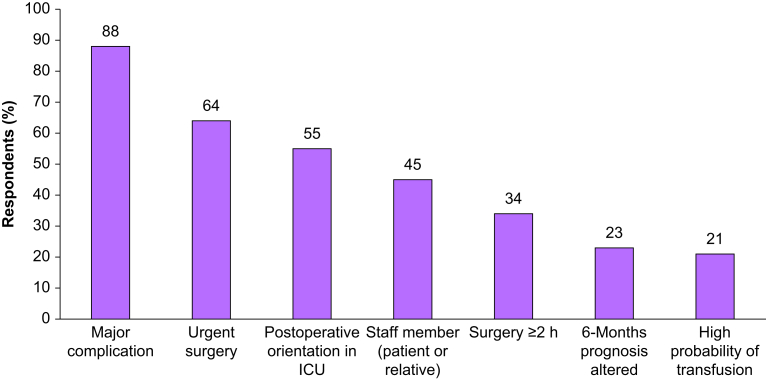
Situations in which information is preferable according to clinicians. ICU, intensive care unit.

Anaesthesiologists were asked in a ‘check all that apply’ question if there were particular situations for which providing information would be important. These situations are presented in Fig. 1.

Sixty-seven percent thought there would be a benefit from provision of information during surgery for the relatives' well-being, whereas a few found benefit for themselves (21%), for patient well-being (18%), and for patient management during the postoperative period (21%). In an open-labelled question, anaesthesiologists reported that giving information during surgery could improve relations between anaesthesiologists and relatives or with the patient themself.

### Reasons for not speaking to relatives

**Fig 2:**
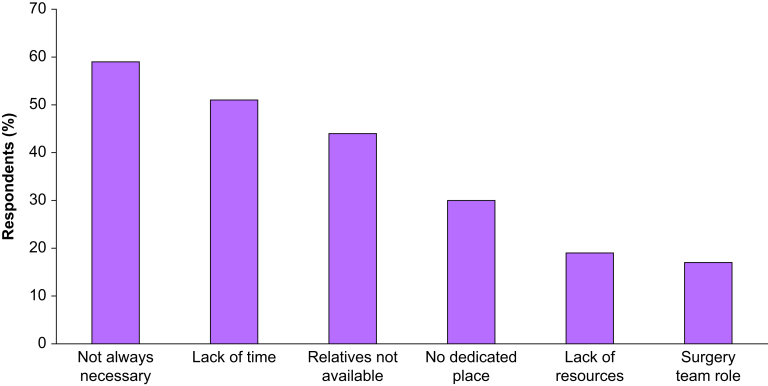
Reasons for not informing relatives during surgery.

Twenty-eight percent of anaesthesiologists did not see any point in providing information to the patients' relatives during surgery. The reasons for which they did not wish to do so are summarised in Fig. 2.

## Discussion

The I-POP survey aimed to assess the rate of information provision to relatives, during surgery, as declared by anaesthesiologists and to identify the factors associated with more frequent communication between anaesthesiologists and relatives. The results suggest that French anaesthesiologists rarely talk to their patient's relatives while surgery is taking place, but also that in some situations it may be necessary to do so. This is the first study, to our knowledge, to provide an overview of current practice on information during surgery by anaesthesiologists to relatives of surgical patients.

Only 8% of anaesthesiologists declared that they provide information during surgery to relatives frequently (i.e. >50% of the time). We found several independent factors associated with providing information frequently: age >50 yr, female gender, and physicians who have a paediatric practice (Table 2). Providing information before surgery more frequently and having a phone number to contact relatives were also associated with providing information during surgery frequently in univariate analysis (Table 1), but were not retained in the final model of the multivariate analysis (Table 2).

A duty of communication is a fundamental pillar in medicine.^19^ We have to inform the patient and ensure their comprehension and consent. By informing relatives, their own comprehension is enhanced and they are more involved in holistic patient care.^20^ A better understanding of the situation will probably improve the relatives' overall confidence in the healthcare team.^21^ It may also reduce their own anxiety and improve their satisfaction.^22^ The patient may also benefit from this holistic care strategy.

Family-centred perioperative care is well implemented for children,^23^ which probably explains why having a paediatric practice was an independent factor associated with providing information during surgery frequently (Table 2). Family-centred care is also more and more studied and optimised in the ICU.^1^ However, data and interventions are lacking for family-centred perioperative care in adults. We present in Supplementary Material 2 the virtuous and vicious circles that can appear with or without family-centred perioperative care, respectively.

Previously, significant differences have been described between male and female physicians, consistent with the results of our study, showing a positive association between female gender and providing information during surgery more frequently.^24^^,^^25^ Similarly, in China, female students tended to have more patient-centred attitudes than male students,^26^ as already demonstrated in other countries.^27^

Being older than 50 yr was also associated with providing information during surgery more frequently. A few studies have previously found a link between patient satisfaction and increasing physician age. In a cohort study of 1342 ophthalmologists,^28^ increasing age was associated with a decreased risk of receiving a patient complaint. However, this association of age and outcome related to patient centredness was inconsistent in the literature.^29^

Anaesthesiologists were asked the reasons why information during surgery was not provided. Major points raised were a lack of time and resources, and the fact that relatives were not available (Fig 2). Some anaesthesiologists also had doubts about the usefulness of such information.

Anaesthesiologists also frequently felt that information during surgery was not useful because of the instability of the clinical situation and potential complications which could occur at any time, the PACU period included. However, recent studies have shown a low rate of serious complications during scheduled surgery. Both the percentage of anaesthesia claims in all claims submitted to National Health Service (NHS) Resolution (1.5% *vs* 2.5%) and the cost of all claims related to anaesthesia (0.7% *vs* 2.4%) have decreased.^30^ Regional anaesthesia (24%), insufficient anaesthesia (20%), and medication administration (20%) were the most prevalent clinical categories.^30^ Claims for central venous catheterisation, cardiac arrest, and airway management continued to be rare but severe and expensive.^30^ These data may also help anaesthesiologists to adapt the information (content and timing) they provide according to the specific clinical setting and the patient's own characteristics, to ensure information validity and relatives' confidence.

Our survey has several limitations. First, as data were collected on the basis of a declarative survey there is necessarily a self-selection bias.^31^ Second, some interesting points could not be addressed in our questionnaire, as we had to limit the number of items (there were 29). We were not able to separate respective information rates from paediatric only and adult only anaesthesiologists, as most anaesthesiologists were providing both paediatric and adult care. It would have been interesting to study more precisely differences in habits and practice between adults and children. Third, we only asked anaesthesiologists (physicians) and did not address information provided by anaesthetist nurses and other operative theatre and surgical wards staff. Fourth, there are probably important differences in relatives' needs and expectations within the same country and between different countries.^32^ Responders were from France, so caution should be exercised when generalising our results. As the questionnaire was openly accessible on the French Society website, and the response rate estimated ∼40%, how our cohort of respondents was representative of anaesthesiologists in France is questionable. In particular, we may suspect that anaesthesiologists interested in communication with relatives were more likely to participate. Fifth, during the Coronavirus Disease 2019 (COVID-19) period, relatives were not present in the hospital. In ICU, the impact of family visitation restrictions was well studied and associated in a qualitative study with clinician emotional exhaustion and emotional distress alongside the negative impact on job satisfaction.^33^ Another study^34^ revealed that among family members of patients hospitalised in the ICU with acute respiratory distress syndrome, COVID-19, compared with other causes of acute respiratory distress syndrome, was significantly associated with increased risk of symptoms of post-traumatic stress disorder at 90 days after ICU discharge. All these points suggest that the restriction of family visitation during the COVID-19 pandemic might be associated with worse prognosis of the relative, and need to be evaluated in operating room setting.

We believe that our survey offers useful suggestions for further work in this field. A research agenda can identify research priorities after the results of our survey. Information given to relatives can certainly be improved and the difficulties expressed in our survey are a starting point to find means to facilitate information provision to relatives by anaesthesiologists in their daily practice. A large international multicentre observational study should be performed to answer the question of daily practices of anaesthesiologists worldwide in real life regarding the informing of relatives, as practices may differ between countries. Further studies could focus on situations of interest, such as urgent surgery or lasting >2 h (Fig 1), for which information during surgery could benefit relatives and the patient in terms of care satisfaction and symptoms of anxiety and benefit the patient in terms of postoperative pain and other clinically relevant outcomes. Information during surgery might be less beneficial in shorter procedures, as the patient is able to contact their relatives by text soon after arrival on the postoperative ward. The usual information strategy could be compared to a wider, more complete and defined ‘information to relatives during surgery’ strategy in a stepped wedge cluster randomised controlled trial. By studying new pathways of information,^35^^,^^36^ taking into account the objections raised by anaesthesiologists such as lack of time and dedicated places (Fig. 2), we could answer the question of the effect of information on stress, anxiety symptoms for relatives and the patient, as part of a multimodal analgesia strategy.^37^

In summary, we report objective data on the frequency of giving information to relatives during surgery, from entry to the operating theatre to release from PACU, by anaesthesiologists in a large number of French districts. Providing information during surgery in >50% of cases was only reported by 8% of anaesthesiologists. We also identified some situations associated with providing information during surgery more frequently and the difficulties which may explain the current rarity of this communication in everyday practice. Using these data, we could implement global care strategies to improve communication with relatives and embed it in our daily work.

## Author's contributions

Conceptualisation: ADJ, NK, MT, CL, MP, YP.

Methodology, software: ADJ.

Interpretation of data: ADJ, NK, JC.

Writing, original draft preparation: ADJ, CP, ST.

Data curation: NK.

Acquisition of data: CP.

Writing, reviewing and editing: NK, JC, MT, CL, MP, YP.

Conception and design, visualisation, investigation, supervision, validation: SJ.

Provided final approval of the version to be published, and agreed to be accountable for all aspects of the work thereby ensuring that questions related to the accuracy or integrity of any part of the work are appropriately investigated and resolved: all authors.

## Declarations of interest

SJ reports receiving consulting fees from Drager, Medtronic, Mindray, Fresenius, Baxter, and Fisher & Paykel. ADJ reports receiving remuneration for presentations from Medtronic, Drager, and Fisher & Paykel. All other authors declare that they have no conflicts of interest.

## Funding

The study is an investigator-initiated trial. The study sponsor is the Montpellier University Hospital, Montpellier, France. There was no industry support or involvement in the study. The funder had no role in the design or conduct of the study, data collection, analysis or interpretation, the writing of the report or in the decision to submit for publication. The corresponding author had full access to all of the data and the final responsibility to submit for publication.

## References

[1] Davidson J.E., Aslakson R.A., Long A.C. (2017). Guidelines for family-centered care in the neonatal, pediatric, and adult ICU. Crit Care Med.

[2] Alkadri J., Aucoin S.D., McDonald B., Grubic N., McIsaac D.I. (2022). Association of frailty with days alive at home in critically ill patients undergoing emergency general surgery: a population-based cohort study. Br J Anaesth.

[3] Engel J.S., Tran J., Khalil N. (2023). A systematic review of perioperative clinical practice guidelines for care of older adults living with frailty. Br J Anaesth.

[4] Fowler A.J., Stephens T.J., Partridge J., Dhesi J. (2022). Surgery in older patients: learning from shared decision-making in intensive care. Br J Anaesth.

[5] Schmocker R.K., Cherney Stafford L.M., Siy A.B., Leverson G.E., Winslow E.R. (2015). Understanding the determinants of patient satisfaction with surgical care using the Consumer Assessment of Healthcare Providers and Systems surgical care survey (S-CAHPS). Surgery.

[6] Blum E.P., Burns S.M. (2013). Perioperative communication and family members' perceived level of anxiety and satisfaction. ORNAC J.

[7] Tylee M.J., Rubenfeld G.D., Wijeysundera D., Sklar M.C., Hussain S., Adhikari N.K.J. (2020). Anesthesiologist to patient communication: a systematic review. JAMA Netw Open.

[8] Trinh L.N., Fortier M.A., Kain Z.N. (2019). Primer on adult patient satisfaction in perioperative settings. Perioper Med (Lond).

[9] Wijeysundera D.N. (2011). Preoperative consultations by anesthesiologists. Curr Opin Anaesthesiol.

[10] Levinson W., Hudak P., Tricco A.C. (2013). A systematic review of surgeon-patient communication: strengths and opportunities for improvement. Patient Educ Couns.

[11] De Jong A., Kentish N., Souppart V., Jaber S., Azoulay E., Preiser Jean Charles, Herridge Margaret, Azoulay Elie (2020). Post-intensive care syndrome.

[12] Kentish-Barnes N., Chevret S., Valade S. (2022). A three-step support strategy for relatives of patients dying in the intensive care unit: a cluster randomised trial. Lancet.

[13] Fumis R.R., Ranzani O.T., Faria P.P., Schettino G. (2015). Anxiety, depression, and satisfaction in close relatives of patients in an open visiting policy intensive care unit in Brazil. J Crit Care.

[14] Hussein M., Pavlova M., Ghalwash M., Groot W. (2021). The impact of hospital accreditation on the quality of healthcare: a systematic literature review. BMC Health Serv Res.

[15] Luz M., Brandão Barreto B., de Castro R.E.V. (2022). Practices in sedation, analgesia, mobilization, delirium, and sleep deprivation in adult intensive care units (SAMDS-ICU): an international survey before and during the COVID-19 pandemic. Ann Intensive Care.

[16] Eysenbach G. (2004). Improving the quality of web surveys: the checklist for reporting results of internet E-surveys (CHERRIES). J Med Internet Res.

[17] Ranganathan P., Pramesh C.S., Aggarwal R. (2017). Common pitfalls in statistical analysis: logistic regression. Perspect Clin Res.

[18] Newgard C.D., Lewis R.J. (2015). Missing data: how to best account for what is not known. JAMA.

[19] Middleton A., Milne R., Robarts L., Roberts J., Patch C. (2019). Should doctors have a legal duty to warn relatives of their genetic risks?. Lancet.

[20] Kentish-Barnes N., Cohen-Solal Z., Morin L., Souppart V., Pochard F., Azoulay E. (2021). Lived experiences of family members of patients with severe COVID-19 who died in intensive care units in France. JAMA Netw Open.

[21] Azoulay E., Pochard F., Chevret S. (2003). Family participation in care to the critically ill: opinions of families and staff. Intensive Care Med.

[22] Azoulay E., Pochard F., Chevret S. (2002). Impact of a family information leaflet on effectiveness of information provided to family members of intensive care unit patients: a multicenter, prospective, randomized, controlled trial. Am J Respir Crit Care Med.

[23] Mendoza B.A., Fortier M.A., Trinh L.N., Schmid L.N., Kain Z.N. (2021). Factors impacting parental and child satisfaction in the perioperative setting. Paediatr Anaesth.

[24] Roter D.L., Hall J.A., Aoki Y. (2002). Physician gender effects in medical communication: a meta-analytic review. JAMA.

[25] Heath J.K., Dine C.J., LaMarra D., Cardillo S. (2021). The impact of trainee and standardized patient race and gender on internal medicine resident communication assessment scores. J Grad Med Educ.

[26] Liu W., Hao Y., Zhao X. (2019). Gender differences on medical students’ attitudes toward patient-centred care: a cross-sectional survey conducted in Heilongjiang, China. PeerJ.

[27] Wahlqvist M., Gunnarsson R.K., Dahlgren G., Nordgren S. (2010). Patient-centred attitudes among medical students: gender and work experience in health care make a difference. Med Teach.

[28] Fathy C.A., Pichert J.W., Domenico H., Kohanim S., Sternberg P., Cooper W.O. (2018). Association between ophthalmologist age and unsolicited patient complaints. JAMA Ophthalmol.

[29] Ajmi S.C., Aase K. (2021). Physicians’ clinical experience and its association with healthcare quality: a systematised review. BMJ Open Qual.

[30] Oglesby F.C., Ray A.G., Shurlock T., Mitra T., Cook T.M. (2022). Litigation related to anaesthesia: analysis of claims against the NHS in England 2008-2018 and comparison against previous claim patterns. Anaesthesia.

[31] Safdar N., Abbo L.M., Knobloch M.J., Seo S.K. (2016). Research methods in healthcare epidemiology: survey and qualitative research. Infect Control Hosp Epidemiol.

[32] Azoulay E., Pochard F., Chevret S. (2004). Half the family members of intensive care unit patients do not want to share in the decision-making process: a study in 78 French intensive care units. Crit Care Med.

[33] McPeake J., Kentish-Barnes N., Banse E. (2023). Clinician perceptions of the impact of ICU family visiting restrictions during the COVID-19 pandemic: an international investigation. Crit Care.

[34] Azoulay E., Resche-Rigon M., Megarbane B. (2022). Association of COVID-19 acute respiratory distress syndrome with symptoms of posttraumatic stress disorder in family members after ICU discharge. JAMA.

[35] Kuizenga M.H., Vereecke H.E.M., Absalom A.R. (2022). Utility of the SmartPilot® View advisory screen to improve anaesthetic drug titration and postoperative outcomes in clinical practice: a two-centre prospective observational trial. Br J Anaesth.

[36] Jiao Y., Xue B., Lu C., Avidan M.S., Kannampallil T. (2022). Continuous real-time prediction of surgical case duration using a modular artificial neural network. Br J Anaesth.

[37] Koschmieder K.C., Funcke S., Shadloo M. (2023). Validation of three nociception indices to predict immediate postoperative pain before emergence from general anaesthesia: a prospective double-blind, observational study. Br J Anaesth.

