# Supplementary material for: Determinants of information provided by anaesthesiologists to relatives of patients during surgical procedures
Source: BJA Open. 2023 Jun 28;7:100205. doi: 10.1016/j.bjao.2023.100205 (PMC10457491; doi:10.1016/j.bjao.2023.100205)
Supplement: Multimedia component 1 [file mmc1.docx]

**I-POP SURVEY**

**Physician’s characteristics**

1. **Age (years):**

1. **Sex:**

M

F

1. **Structure of exercise:**

Teaching Hospital

General Hospital

Medical Clinic

ESPIC

Alternative proposition

1. **Region / District of exercise:**

Auvergne-Rhône-Alpes

Bourgogne-Franche-Comté

Bretagne

Centre-Val de Loire

Corse

Grand Est

Hauts-de-France

Île-de-France

Normandie

Nouvelle Aquitaine

Occitanie

Pays de la Loire

Provence-Alpes-Côte d’Azur

Régions d’Outre Mer

1. **Function:**

Resident

Senior Doctor / Assitant

Attached Practioner Hospital Practioner

Senior Lecturer University Professor

Private Practioner

1. **Experience:**

Resident sem 1-5

Resident sem 6-10

Anesthesiologist graduated < 5yrs

Anesthesiologist graduated 5-10yrs

Anesthesiologist graduated 10-20yrs

Anaesthesiologist graduated > 20 ans

1. **Activity field:**

Adult > 50% Paediatrics > 50%

General surgical unit Specialized surgical unit

Surgical unit specialisation: …………………………………………………….

**PRE OPERATIVE STANDARD OF CARE : ANAESTHESIOLOGIST PRACTICE HABITS**

1. **Pre operative interview with family of surgical patient: (single answer question)**

Never 0% of cases

Sometimes < 50% of cases

Often > 50 % of cases

Always 100 % of cases

If « Never », then go forward question 9

1. **Interview modality: (check all that apply question)**

Anesthetic consultation

Pre anesthetic visit

Operative room waiting room

Emergency Department

Phone call

Alternative proposition

1. **Pre operative information to relatives content: anaesthetic protocol: (single answer question)**

Never 0% of cases

Sometimes < 50% of cases

Often > 50 % of cases

Always 100 % of cases

1. **Pre operative information to relatives content: anaesthetic risk: (single answer question)**

Never 0% of cases

Sometimes < 50% of cases

Often > 50 % of cases

Always 100 % of cases

1. **Pre operative information to relatives content: post operative potential complications: (single answer question)**

Never 0% of cases

Sometimes < 50% of cases

Often > 50 % of cases

Always 100 % of cases

1. **Pre operative information to relatives content: post operative pain management: (single answer question)**

Never 0% of cases

Sometimes < 50% of cases

Often > 50 % of cases

Always 100 % of cases

1. **Relatives’ further questions? (check all that apply question)**

Patient’s pain management

Patient’s anxiety management

Anaesthetic protocol

Anaesthetic risk

Surgical gesture

Post operative complications

Logistical issues, time management

Spatial arrangement (wards/operative room)

Alternative proposition(s), please specify  ……………………………………………………..

1. **Situation(s) for which an information for relatives is preferrable in pre operative period (single answer per question)**
   1. **Depending on the type of surgery (ex: carcinological surgery, paediatric…)**

Yes

Please specify: …………………………………………………………………………………

No

- 1. **Depending on emergency circumstances (urgent surgery vs scheduled surgery)**

Yes

No

- 1. **Depending on surgery length (short surgical gesture <2h vs long)**

Yes

No

- 1. **Depending on anaesthetic risk (high risk vs low risk)**

Yes

No

- 1. **Depending transfusion risk factors (high probability vs low probability)**

Yes

No

- 1. **Depending on patient’s estimated prognosis at 6 months (bad vs good prognosis)**

Yes

No

- 1. **Depending on patient’s post operative ward orientation**

Ambulatory

Surgical ward

Intensive care unit

Intensive care unit – resuscitation unit

- 1. **The patient or his relatives work(s) in a healthcare structure**

Yes

No

1. **A telephone number is provided to relatives or a contact person is designed to join by families ?** **(single answer question)**

Never 0% of cases

Sometimes < 50% of cases

Often > 50 % of cases

Always 100 % of cases

1. **Support person / Contact person identity and phone number are gathered ? (single answer question)**

Never 0% of cases

Sometimes < 50% of cases

Often > 50 % of cases

Always 100 % of cases

1. **In your opinion, is there any interest to inform surgical patient family during pre operative period ? (check all that apply question)**

It could benefit from the anaesthesiologist himself

It could benefit from family well-being

It could benefit from the patient pre-operatively

It could benefit from the patient post-operatively

None

**DURING INTRA-OPERATIVE AND IMMEDIATE POST-OPERATIVE PERIODS**

**1. Intra-operative family’s information of surgical patient: (single answer question)**

Never 0% of cases

Sometimes < 50% of cases

Often > 50 % of cases

Always 100 % of cases

If « Never » is chosen, then, pass to question number 13.

**2. Who deliver intra-operative information ? (check all that apply question)**

Anaesthesiologist

Resident in Anaesthesia

Anaesthesia nurse

Surgeon

Resident in surgery

Operating nurse

Dedicated person not present in operating room

**3. Information is provided together with the surgery team? (single answer question)**

Never 0% of cases

Sometimes < 50% of cases

Often > 50 % of cases

Always 100 % of cases

**4. Intra-operative information mode? (check all that apply question)**

Phone call

Interview in a corridor

Interview in the waiting room

Interview in a dedicated study

Interview in the patient’s room

Other, please specify:…………………………………………………………..

**5. Which moment is preferentially chosen ? (check all that apply question)**

After anaesthetic induction

Half time surgery

After surgical ablation

At surgical closure

Exit from operating room to PACU

Occurrence of a complication

According to informer availability

Other , please specify: ……………………………………………………………..

**6. In absence of intra operative complication which topics are raised? (check all that apply question)**

Surgery progress

Patient’s clinical setting

Information about time to release of operating room and PACU

Patient’s postoperative ward

Information about waiting room

Other , please specify: …………………………………………………………

**7. Relatives’ further questions ? (check all that apply question)**

About surgery gesture

About anaesthesia

About potential complications

About post-operative period

About waiting room

About length of surgery

About length of stay in PACU

Never

1. **The patient is willing to ask for information for his family (single answer question)**

Never 0% of cases

Sometimes < 50% of cases

Often > 50 % of cases

Always 100 % of cases

1. **Families are willing to be informated during intra-operative period (single answer question)**

Never 0% of cases

Sometimes < 50% of cases

Often > 50 % of cases

Always 100 % of cases

1. **Are there situations for which you would rather inform families during intra-operative period ? (single answer question for each item)**
   1. Type of surgery (oncological, paediatrics..)

Yes

If yes, please specify: …………………………………………………………..

No

- 1. Urgent surgery (urgent surgery versus scheduled surgery)

Yes

No

- 1. According to surgery length (long surgery > 2h versus short surgical procedures)

Yes

No

- 1. According to transfusion risks (high versus low risk)

Yes

No

- 1. In the case of the occurrence of a major complication

Yes

No

- 1. According to patient estimated prognosis at 6 months

Yes

No

- 1. According to patient postoperative orientation (ambulatory, surgical ward, intensive care unit)

Yes

No

- 1. The patient or the relative is a caregiver

Yes

No

**11. In your opinion, is there any interest to inform surgical patient family during intra operative period ? (check all that apply question)**

It could benefit from the anaesthesiologist himself

It could benefit from family well-being

It could benefit from the patient pre-operatively

It could benefit from the patient post-operatively

None

1. **If families are not informed, which is/are the reason(s) ? (check all that apply question)**

Lack of time

Lack of material resources

No dedicated place

Surgical team role

Not always necessary

Relatives not available

Other , please specify: …………………………………………………….

1. **Do relatives have a simply mean to obtain information during intra-operative period ?** **(check all that apply question)**

Telephone number to join

Somewhere to request for information

Other , please specify : ……………………………………………………….

None

1. **Are there service standards in intra operative family information in your ward ? (single answer)**

Yes

No

If yes, could you please send it by email » «
