# Supplementary material for: Determinants of information provided by anaesthesiologists to relatives of patients during surgical procedures
Source: BJA Open. 2023 Jun 28;7:100205. doi: 10.1016/j.bjao.2023.100205 (PMC10457491; doi:10.1016/j.bjao.2023.100205)
Supplement: Multimedia component 2 [file mmc2.docx]

SUPPLEMENTARY MATERIAL 2

Determinants of information provided by anaesthesiologists to relatives of patients during surgical procedures

Supplementary Figure 1. Virtuous circle that can appear with family-centred perioperative care


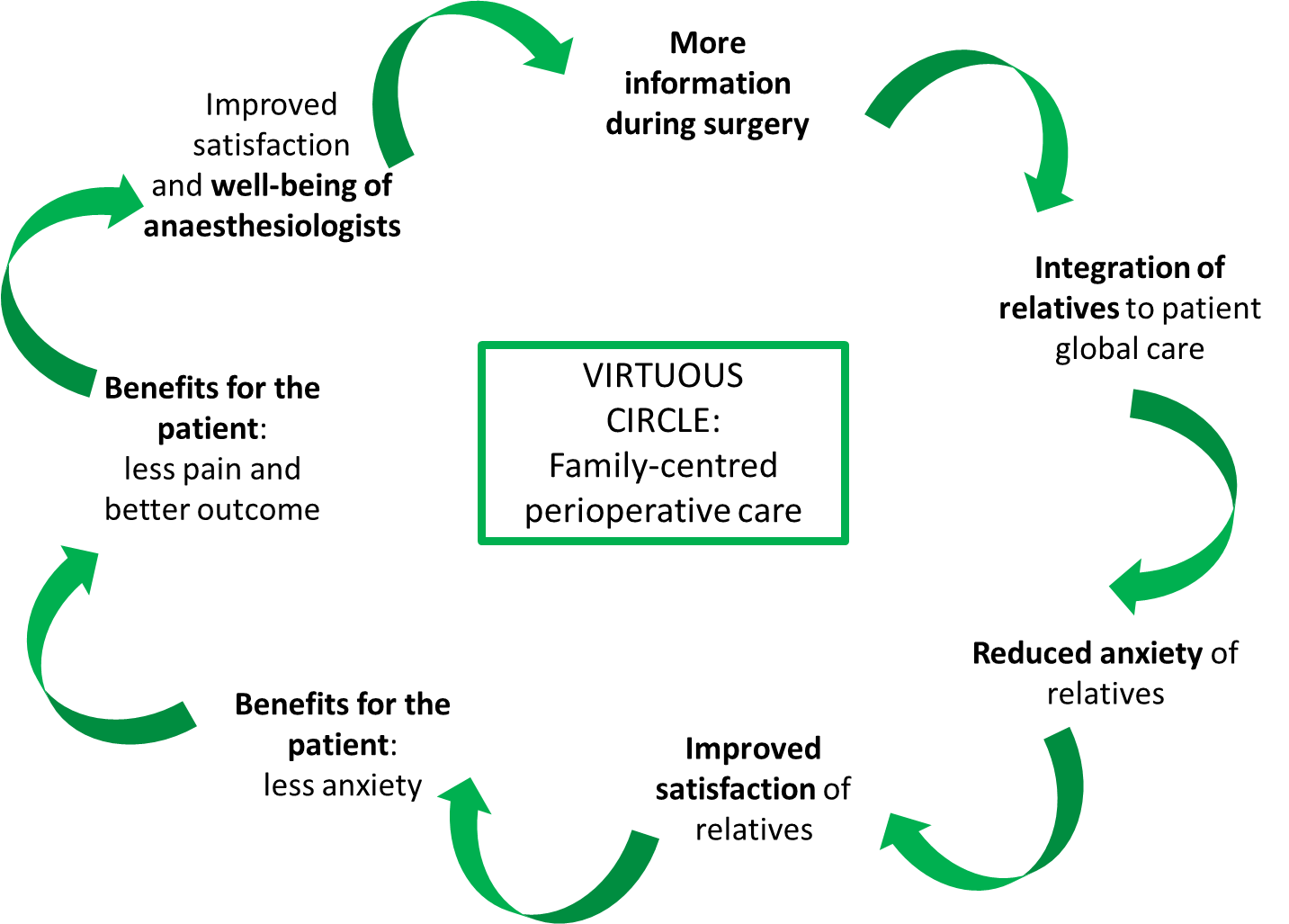


Supplementary Figure 2. Vicious circle that can appear without family-centred perioperative care


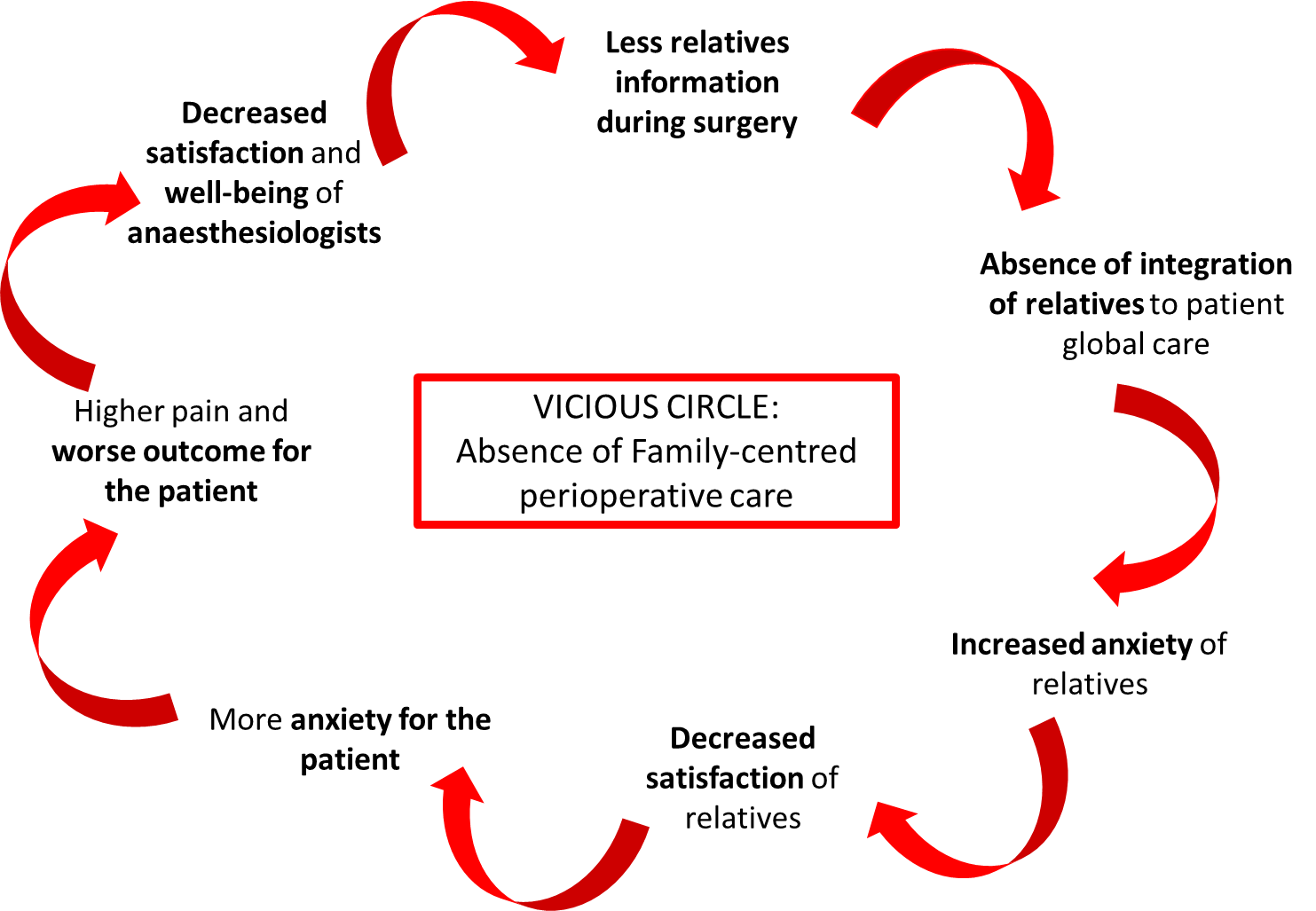


Table 1. Factors associated with providing information during surgery more frequently to relatives in univariate analysis

| Characteristics | Information in more than 50% of cases  n=49 | Information in less than 50% of cases n=558 | P value |
| --- | --- | --- | --- |
| Age | 55 (44-62) | 46 (35-59) | < 0.001 |
| Age > 50 years, n (%) | 31 (63) | 226 (41) | 0.002 |
| Male gender, n (%) | 22 (45) | 297 (53) | 0.263 |
| Structure of exercise, n (%) |  |  | 0.969 |
| Teaching Hospital | 20 (41) | 216 (39) |  |
| General Hospital | 9 (18) | 117 (21) |  |
| Medical Clinic | 17 (35) | 186 (33) |  |
| Other | 3 (6) | 39 (7) |  |
| Function, n (%) |  |  | 0.062 |
| Resident | 0 (0) | 35 (6) |  |
| Senior Doctor / Assistant | 0 (0) | 43 (7) |  |
| Attached Practioner | 2 (4) | 20 (4) |  |
| Hospital Practioner | 29 (59) | 257 (46) |  |
| Senior Lecturer | 0 (0) | 4 (1) |  |
| University Professor | 1 (2) | 4 (1) |  |
| Private Practitioner | 17 (35) | 195 (35) |  |
| Experience, n (%) |  |  | 0.027 |
| Resident | 0 (0) | 35 (6) |  |
| Anaesthesiologist < 5years | 4 (8) | 96 (17) |  |
| Anaesthesiologist 5-10years | 9 (18) | 88 (16) |  |
| Anaesthesiologist 10-20years | 6 (12) | 109 (20) |  |
| Anaesthesiologist > 20years | 30 (61) | 230 (41) |  |
| Activity field, n (%) |  |  |  |
| Adult > 50% | 34 (69) | 477 (85) | < 0.001 |
| Paediatrics > 50% | 15 (31) | 81 (15) | 0.003 |
| Information frequency before surgery, n (%) |  |  | < 0.001 |
| Never | 0 (0) | 14 (3) |  |
| Sometimes | 10 (20) | 337 (60) |  |
| Often | 21 (43) | 167 (30) |  |
| Always | 18 (37) | 40 (7) |  |
| Relative identified in the medical record, n (%) |  |  | 0.027 |
| Never | 4 (8) | 59 (11) |  |
| Sometimes | 5 (10) | 153 (27) |  |
| Often | 8 (17) | 93 (17) |  |
| Always | 32 (65) | 253 (45) |  |

Table 2. Factors associated with providing information during surgery more frequently to relatives in multivariate analysis

| Characteristics | OR | 95% CI | P-value |
| --- | --- | --- | --- |
| Age > 50 years | 2.830 | 1.5-5.3 | < 0.01 |
| Female gender | 1.880 | 1.0-3.5 | 0.047 |
| Paediatric activity field | 2.487 | 1.3-4.8 | < 0.01 |

95% CI, 95% confidence interval; OR, odds ratio
